# Supplementary material for: Magnon-phonon Fermi resonance in antiferromagnetic CoF2
Source: Nat Commun. 2024 Jun 28;15:5472. doi: 10.1038/s41467-024-49716-w (PMC11213879; doi:10.1038/s41467-024-49716-w)
Supplement: Supplementary file 1 — Supplementary Information [file 41467_2024_49716_MOESM1_ESM.pdf]

Supplementary: Magnon-phonon Fermi resonance in antiferromagnetic CoF_2

Thomas W.J. Metzger[1*], Kirill A. Grishunin[1], Chris Reinhoffer[2], Roman M. Dubrovin[3], Atiqa Arshad[4], Igor Ilyakov[4], Thales V.A.G. de Oliveira[4], Alexey Ponomaryov[4], Jan-Christoph Deinert[4], Sergey Kovalev[4], Roman V. Pisarev[3], Mikhail I. Katsnelson[1], Boris A. Ivanov[1], Paul H. M. van Loosdrecht[2], Alexey V. Kimel[1] and Evgeny A. Mashkovich[2*]

[1]Institute for Molecules and Materials, Radboud University, Heyendaalseweg 135, Nijmegen, 6525 AJ, The Netherlands.
[2]Institute of Physics II, University of Cologne, Zuelpicher Straße 77, Cologne, 10587, Germany.
[3]Ioffe Institute, Russian Academy of Sciences, St. Petersburg, 194021, Russia.
[4]Institute of Radiation Physics, Helmholtz-Zentrum Dresden-Rossendorf, Bautzner Landstraße 400, Dresden, 01328, Germany.

*Corresponding author(s). E-mail(s): thomas.metzger@ru.nl; mashkovich@ph2.uni-koeln.de;

Appendix A Additional experimental data

To estimate the magnon and phonon Gilbert damping factors, we employ the fitting function

$$y = A + B\sqrt{\frac{1}{(x^2-\omega_i^2)^2+4\zeta_i^2x^2}} \tag{A1}$$

to the spectra corresponding the highest magnon and phonon responses, see Fig. A1(a-b). Here, A represents an offset, B is the spectral amplitude and ω_i

are the respective magnon and phonon center frequencies. We extract $\zeta_{\mathrm{m}}/2\pi \approx 10$ GHz and $\zeta_{\mathrm{ph}}/2\pi < 5$ GHz, respectively. However, as can be seen from Fig. A1(c), the phonon is practically not damped for our time range of 130 ps. Hence, we assume a Gilbert damping of $\zeta_{\mathrm{ph}}/2\pi = 0$ for the phonon in our simulation.

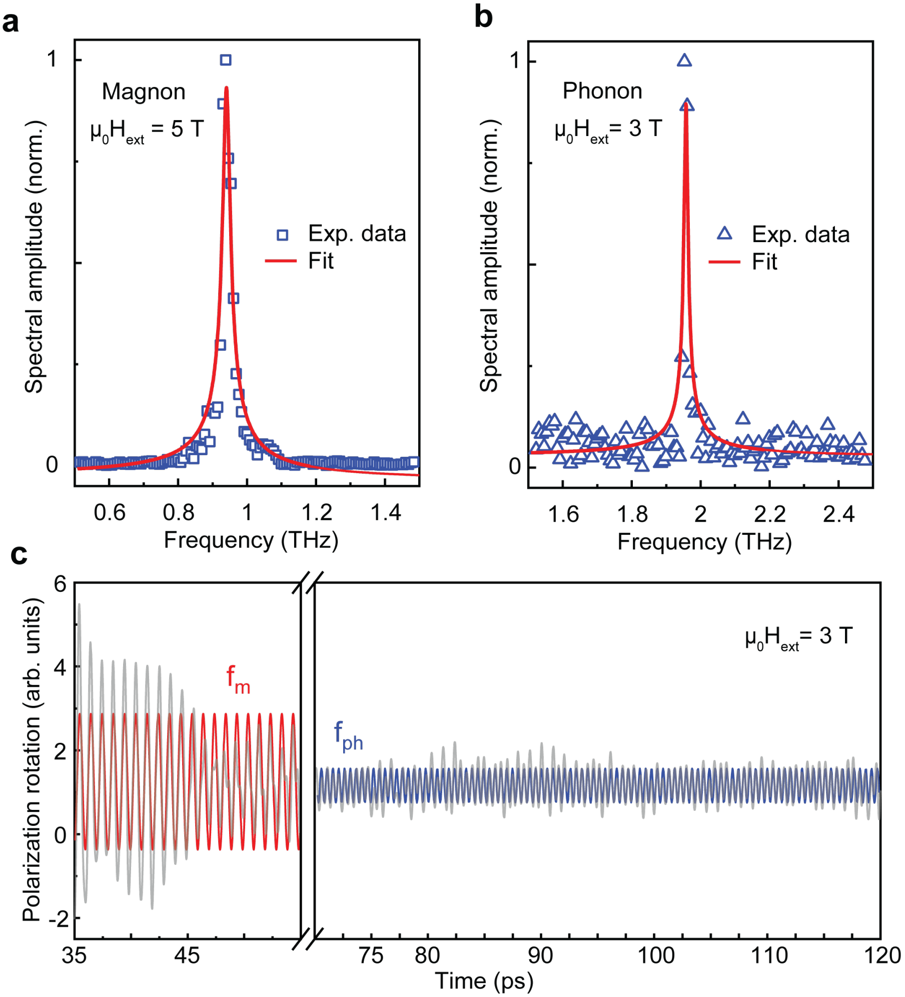

Fig. A1 (a-b) Extraction of the Gilbert damping parameters by fitting Eq. A1 (red line) to the magnon and the phonon the experimental data points (blue). Spectral amplitude (blue data points) of (a) magnon at $\mu_0 H_{\mathrm{ext}} = 5$T and (b) phonon at $\mu_0 H_{\mathrm{ext}} = 3$ T of external magnetic field, where the respective mode is pronounced strongest. (c) Effect of magnon and phonon damping. Time domain data is filtered by a lowpass filter with a cutoff frequency of 2.5 THz to remove high frequency noise for $\mu_0 H_{\mathrm{ext}} = 3$ T (grey line). Sinusoidal fits of the magnon (red) and the phonon (blue) responses.

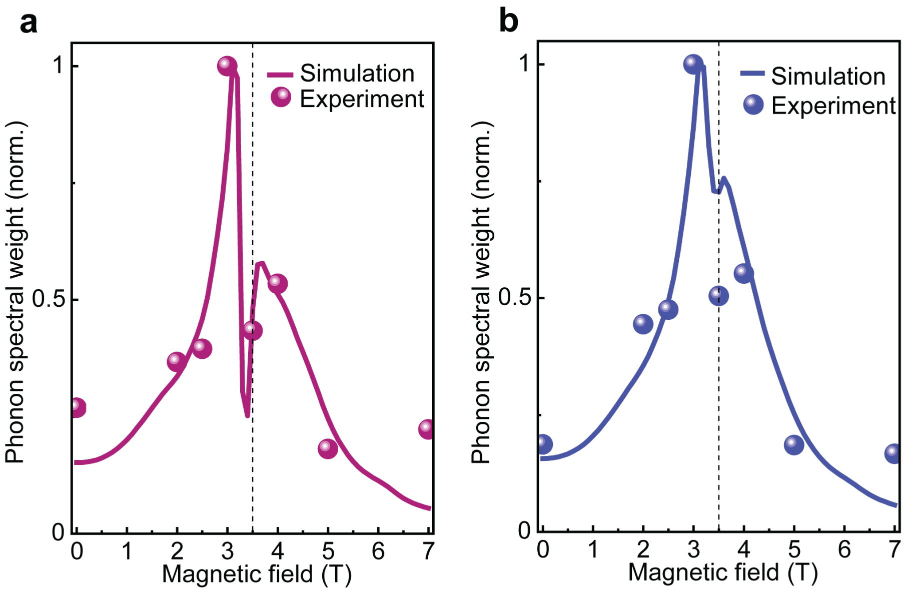

Fig. A2 Phonon spectral weight as a function of external magnetic field. Simulation parameters are $\zeta_{\text{ph}}/2\pi = 0$ GHz and $\alpha = 7$. The frequency range for extraction of the phonon weight is 1.9 - 2.0 THz. The time windows for Fourier transformation are set to (a) 70 - 120 ps and (b) -10 - 120 ps. We note that (b) is a duplicate of main text Fig. 3(b).

As shown in Fig. A1(c), the magnon amplitude dominates from -10 to 70 ps whereas for the range 70 - 120 ps, the dynamics are clearly dominated by the phonon. Moreover, the probe polarization rotation might be sensitive directly to THz pulse magnetic or electric fields via magneto-optic sampling [1] or non-linear electro-optical Kerr effect [2], respectively. To exclude these effect contributing on the time scale of our THz pump pulse, we selected a narrowed time window from 70 to 120 ps to perform Fourier transformation. Using the same procedure as described earlier, the phonon weight vs the external magnetic field is extracted, see Fig. A2(a). In particular, we notice the more pronounced dip of the extracted phonon weight in compassion with the full time domain range shown in Fig. A2(b). Overall, better agreement between the simulation and the experiment is shown in Fig. A2(a) for the narrowed time window. This confirms our hypothesis that above mentioned contributions are indeed influencing the response on the time scale of THz pulse duration.

Appendix B Theoretical consideration

We introduce the Lagrangian $\mathcal{L}$ accounting for the magnon, the phonon and the coupling between them following the sigma model [3]

$$\begin{aligned}\mathcal{L} = \frac{1}{2}\dot{\boldsymbol{l}}^2 + \frac{1}{2}\dot{\theta}_{\rm ph}{}^2 - \frac{1}{2}\omega_0^2\left(l_x^2 + l_y^2\right) - \frac{1}{2}\omega_{\rm ph}{}^2\theta_{\rm ph}{}^2 \\ -\gamma\left(\boldsymbol{H}\cdot[\boldsymbol{l}\times\dot{\boldsymbol{l}}]\right) - \frac{1}{2}\gamma^2\left(\boldsymbol{H}\cdot\boldsymbol{l}\right)^2 - \Phi,\end{aligned} \tag{B2}$$

where the magnon dynamics are introduced via a normalized Néel vector $\boldsymbol{l} = \frac{1}{2M_0}(\boldsymbol{M}_1 - \boldsymbol{M}_2)$ with the sublattice magnetizations $\boldsymbol{M}_1$ and $\boldsymbol{M}_2$. $\theta_{\rm ph}$ is the generalized B_{1g} phonon coordinate. Additionally, $\boldsymbol{l}$ and $\theta_{\rm ph}$ are normalised on the corresponding magnon and phonon masses. The upper dot denotes the time derivative. $\omega_0 = 2\pi f_0 = \gamma\sqrt{H_A H_E}$ is the zero magnetic field magnon frequency and $\omega_{\rm ph}$ is the phonon frequency. The $\boldsymbol{H} = \boldsymbol{h}_{\rm THz} + \boldsymbol{H}_{\rm ext}$ consists of an external magnetic field $\boldsymbol{H}_{\rm ext}$ and a magnetic field of a THz pump pulse $\boldsymbol{h}_{\rm THz}$. THz pulse magnetic field is in the sample plane $\boldsymbol{h}_{\rm THz} = (h_x, h_y, 0)$. The rule of Fermi resonance symmetry implies that the B_{1g} phonon symmetry $(x^2 - y^2)$ should follow the symmetry of the double magnon excitation. Hence, the corresponding nonlinear term can be introduced in Lagrangian as $\Phi = -\alpha(l_x^2 - l_y^2)\theta_{\rm ph}$, where α represents the nonlinear coupling constant between the magnon and the phonon subsystems [4].

We consider $\boldsymbol{H}_{\rm ext} \parallel z$. Taking into account that perturbations l_x and l_y are small and thus l_z can be present as $l_z = \sqrt{1 - (l_x^2 + l_y^2)/2}$. In this case the Lagrangian will simplify to

$$\begin{aligned}\mathcal{L} = \frac{1}{2}\left(\dot{l}_x^2 + \dot{l}_y^2\right) + \frac{1}{2}\dot{\theta}_{\rm ph}{}^2 - \frac{1}{2}\left(\omega_0^2 - \gamma^2 H_{\rm ext}^2\right)\left(l_x^2 + l_y^2\right) \\ -\frac{1}{2}\omega_{\rm ph}^2\theta_{\rm ph}{}^2 - \gamma H_{\rm ext}\left(l_x\dot{l}_y - l_y\dot{l}_x\right) - \gamma\left(h_x\dot{l}_y - h_y\dot{l}_x\right) \\ -\alpha\theta_{\rm ph}\left(l_x^2 - l_y^2\right).\end{aligned} \tag{B3}$$

Following the theoretical formalism of [5], equations describing magnetization dynamics of a collinear antiferromagnet can be derived from the Lagrangian $\mathcal{L}$ and the dissipative Rayleigh function

$$R = \zeta_m\left(\dot{l}_x^2 + \dot{l}_y^2\right) + \zeta_{ph}\dot{\theta}_{\rm ph}{}^2. \tag{B4}$$

Substitution into Lagrange-Euler equations

$$\frac{d}{dt}\frac{\partial\mathcal{L}}{\partial\dot{q}} - \frac{\partial\mathcal{L}}{\partial q} = -\frac{\partial R}{\partial\dot{q}}\ (q = l_x,\, l_y \text{ or } \theta_{\rm ph}), \tag{B5}$$

results in a system of three coupled equations describing dynamics of the magnon and the phonon subsystems accounting for the nonlinear coupling between them.

$$\frac{d^2 l_x}{dt^2} + 2\zeta_{\mathrm{m}} \frac{dl_x}{dt} + \left(\omega_0^2 - \gamma^2 H_{\mathrm{ext}}{}^2\right) l_x - 2\gamma H_{\mathrm{ext}} \frac{dl_y}{dt} + 2\alpha\theta_{\mathrm{ph}} l_x = \gamma \frac{h_y}{dt} \tag{B6}$$

$$\frac{d^2 l_y}{dt^2} + 2\zeta_{\mathrm{m}} \frac{dl_y}{dt} + \left(\omega_0^2 - \gamma^2 H_{\mathrm{ext}}{}^2\right) l_y + 2\gamma H_{\mathrm{ext}} \frac{dl_x}{dt} - 2\alpha\theta_{\mathrm{ph}} l_y = -\gamma \frac{h_x}{dt} \tag{B7}$$

$$\frac{d^2 \theta_{ph}}{dt^2} + 2\zeta_{\mathrm{ph}} \frac{d\theta_{\mathrm{ph}}}{dt} + \omega_{\mathrm{ph}}{}^2 \theta_{\mathrm{ph}} = -\alpha \left(l_x^2 - l_y^2\right), \tag{B8}$$

Neglecting the magnon dissipation $\gamma_{\mathrm{m}} = 0$ and the nonlinear coupling α, the denominator of Eqs. B6-B7 determines two circular polarized magnon eigenstates $l_x \pm i l_y$ with corresponding frequencies.

$$\omega_{\mathrm{m}} = \omega_0 \pm \gamma H_{\mathrm{ext}} \tag{B9}$$

The resulting coupled equations are shown in the main text, see Eqs. 1-3.

Appendix C Details of numerical simulation

Firstly, we select a weak coupling regime with $\alpha = 0.07$ and study the effect of tuning the magnon damping, see Fig. C3(a). We find that for $\zeta_{\mathrm{m}}/2\pi = 10$ GHz, the peak position corresponds to our experimental peak position observed at 3 T, see main text Fig 3(b). For $\zeta_{\mathrm{m}}/2\pi = 1$ GHz, the peak is substantially sharper and moves to higher external magnetic field, closer to the frequency matching condition of $2\mathrm{f_m} = \mathrm{f_{ph}}$. This behaviour resembles the case of Fermi resonance in the CW-regime [6]. For $\zeta_{\mathrm{m}}/2\pi = 100$ GHz, the peak is extremely broad and predominantly originates from virtual state excitation as magnon coherent dynamics cannot be formed in this strongly damped regime. Thus, we select $\zeta_{\mathrm{m}}/2\pi = 10$ GHz for our simulation which is in excellent agreement with our experimental data.

Secondly, we tuned the phonon damping while fixing the coupling constant for strong coupling ($\alpha = 7$) and the magnon damping to $\zeta_{\mathrm{m}}/2\pi = 10$ GHz, see Fig. C3(b). Evidently, for $\zeta_{\mathrm{ph}}/2\pi \leq 0.5$ GHz a clear dip centered around $\mu_0 \mathrm{H_{ext}} = 3.5$ T appears. For an increased phonon damping of $\zeta_{\mathrm{ph}}/2\pi \geq 5$ GHz, this dip disappears. This reflects that the effect of strong coupling in the pulsed regime is highly time-dependent, revealing the significance of the life times of two magnons and a phonon as crucial parameters. Low intrinsic damping enhances the nonlinear coupling effect but also corresponds to the formation of this effect on a very long-time scale. Consequently, studying the dynamics substantially after the initial excitation will lead to a better resolved coupling effect. This observation aligns with our conclusions in Section A, where both simulated and experimental phonon weight in a narrowed time window show much pronounced asymmetry, see Fig. A2.

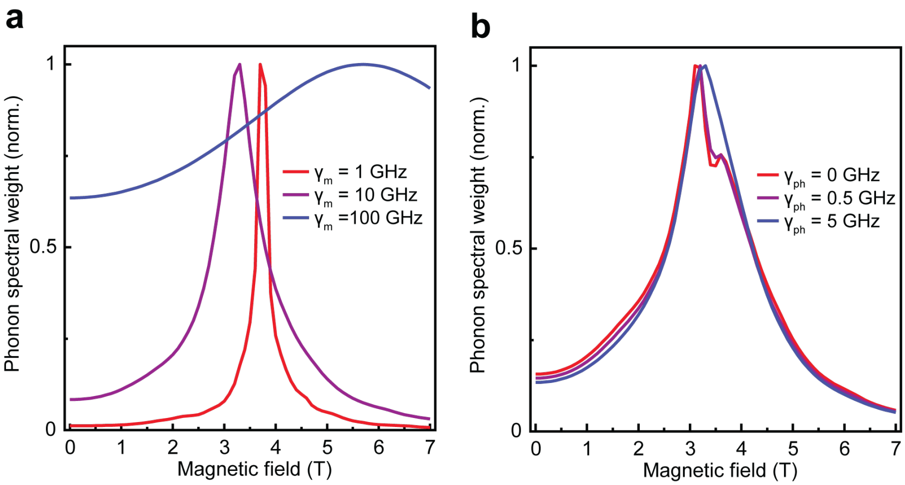

Fig. C3 Simulation parameters. (a) Phonon weight for low coupling regime $\alpha = 0.07$ and phonon damping $\zeta_{\rm ph}/2\pi = 0$ GHz while the magnon damping is varied. (b) Phonon weight for strong coupling regime $\alpha = 7$ and magnon damping $\zeta_{\rm m}/2\pi = 10$ GHz while phonon damping is varied.

References

[1] Riordan, J.A., Sun, F.G., Lu, Z.G., Zhang, X.-C.: Free-space transient magneto-optic sampling. Applied Physics Letters **71**(11), 1452–1454 (1997)

[2] Hoffmann, M.C., Brandt, N.C., Hwang, H.Y., Yeh, K.-L., Nelson, K.A.: Terahertz Kerr effect. Applied Physics Letters **95**(23), 231105 (2009)

[3] Davydova, M.D., Zvezdin, K.A., Kimel, A.V., Zvezdin, A.K.: Ultrafast spin dynamics in ferrimagnets with compensation point. Journal of Physics: Condensed Matter **32**(1), 01–01 (2020)

[4] Breitenberger, E., Mueller, R.D.: The elastic pendulum: A nonlinear paradigm. Journal of Mathematical Physics **22**(6), 1196–1210 (1981)

[5] Galkina, E.G., Ivanov, B.A.: Dynamic solitons in antiferromagnets (Review Article). Low Temperature Physics **44**(7), 618–633 (2018)

[6] Barsukov, I., Lee, H.K., Jara, A.A., Chen, Y.-J., Gonçalves, A.M., Sha, C., Katine, J.A., Arias, R.E., Ivanov, B.A., Krivorotov, I.N.: Giant nonlinear damping in nanoscale ferromagnets. Science Advances **5**(10), 6943 (2019)
